# Supplementary material for: COVID-19 vaccines and mental distress
Source: PLoS One. 2021 Sep 8;16(9):e0256406. doi: 10.1371/journal.pone.0256406 (PMC8425550; doi:10.1371/journal.pone.0256406)
Supplement: S1 Appendix — (PDF) [file pone.0256406.s001.pdf]

S1 Appendix. Effects as a function of time elapsed since first vaccination

We estimate equation SM1, where $Days_{it}$ equals the number of days elapsed since first receiving a vaccine dose (and zero for those who have not been vaccinated by time *t*). β_0 measures the initial impact of receiving the first dose, while β_d measures possible additional effects with the passage of time.

$$Y_{it} = \alpha_i + \tau_t + \beta_0 Vacc_{it} + \beta_d Days_{it} + \varepsilon_{it} \quad \text{(SM1)}$$

A negative coefficient for β_d would indicate a strengthening of the vaccine's mental health impacts, whereas a positive coefficient would indicate decay of such impact. For all four outcomes, the estimated coefficient β_d is negative, but statiscally different from zero at the 5% level only for *severe mental distress*.

Table S.M 1. Time elapsed since first dose,

	PHQ-4 Score (standardized)	Mild mental distress or higher[1]	Moderate mental distress or higher[2]	Severe mental distress[3]
Has vaccine	-0.030***	-0.009*	-0.003	-0.004
Standard error	(0.011)	(0.006)	(0.004)	(0.003)
Days elapsed since vaccination (per 100 days)	-0.035	-0.070	-.004	-0.017**
Standard error	(0.030)	(0.016)	(0.012)	(0.008)
Constant	1.961***	0.291***	0.111***	0.045***
Observations	157,082	157,082	157,082	157,082
R-squared	0.722	0.617	0.529	0.506

Respondent fixed effects and survey-wave dummies included in the regression. [1] *Mild mental distress or higher* is an indicator variable that takes the value of one if PHQ-4 is equal to or higher than three and 0 otherwise; [2] *Moderate mental distress or higher* is an indicator variable that takes the value of one if PHQ-4 is equal to or higher than six and 0 otherwise; [3] *Severe mental distress* is an indicator variable that takes the value of one if PHQ-4 is equal to or higher than six and 0 otherwise. Standard errors clustered at the individual level *** p-value<0.01, ** p-value<0.05, * p-value<0.1
