# Supplementary material for: COVID-19 vaccines and mental distress
Source: PLoS One. 2021 Sep 8;16(9):e0256406. doi: 10.1371/journal.pone.0256406 (PMC8425550; doi:10.1371/journal.pone.0256406)
Supplement: Attachment — Submitted filename: Response to Reviewers.docx [file pone.0256406.s002.docx]

We are deeply grateful for your and the referee’s review and comments. We have revised the paper accordingly and believe it has substantially improved. In particular, per your first comment, we have now better situated the study in the relevant literature.

Below, please find our responses to the individual comments.

I. **Response to the Editor’s comments**

An expert in this field and I have reviewed your submission. We both agree that your contribution may add something to the literature. However, there are some essential corrections needed before I reevaluate your contribution. Please pay attention to all the comments made by the reviewer and answered them using a point-by-point response letter. Apart from his comments, I would like you to consider my following comments as well.

1. **There is a growth of literature on vaccine hesitancy and the relationship between vaccine and mental distress. However, the present study does not provide a comprehensive literature review in this content. The authors are thus recommended to consult the following references to strengthen their literature review.**

*We agree the original submission was lacking on this respect. We have now better situated the study in the relevant literature, making the connection to the literatures on vaccine hesitancy and mental health effects of COVID-19. For context, it is particularly relevant to show that fear of COVID-19, as well as anxiety in general, are predictors of willingness to vaccinate. Page 4 now discusses this literature, which includes all but one of the references suggested by you as well as a number of other references that we deemed relevant.*

2. **The meaning of "Fraction" on Table 1 is unclear. Moreover, what is (1) and (2) in Table 1? Please do not report p at 0.000 because p-value can only be very small but never be 0. Use p < 0.001 instead. The values in the columns of "Never Vaccinated" and "Ever Vaccinated" in Table 1 are unclear what they are.**

*We made the change to use percentages instead of fractions which makes Table 1 clearer in our opinion. We also changed the reporting of p-values to p<0.0001 when applicable, and added the missing column numbers –(1) and (2)- to make the table clearer. With these changes, we hope the contents of Table 1 are now clear.*

3. **In Table 2, please define what "mild mental distress or higher", "moderate mental distress or higher", and "severe mental distress" are. It is unclear what "mean dependent variable" is in Table 2.**

*We now define these variables in the note to Table 2.*

4. **In Table 3, please also define what "mild mental distress or higher", "moderate mental distress or higher", and "severe mental distress" are.**

*We now define these variables in the note to Table 3.*

Reviewers' comments:

Reviewer's Responses to Questions

**Comments to the Author**

**Reviewer #1: Q1: In paragraph 1 of introduction part, the author mentioned the dynamic of mental distress experienced of people due to COVID-19 which is up and down. It would be better to explain more why this fluctuated scheme could happen.
the authors may include the second paragraph into the first paragraph.**

*We added a discussion about a possible explanation for the fluctuation (economic deterioration and recovery) and cited a more extensive literature (page 4).*

**Q2: It is questioned that how the authors convinced the readers that vaccine really can improve well-being and mental health? How the vaccine uptake may reduce stress and alleviate fears of passing a COVID-19 infection along to others in social settings?
There is no citation at all for the 3rd par which talk about vaccine intake**

*We have added a discussion of the literature with the corresponding citations. In particular, we cited literature that showed that people with more anxiety are more likely to be willing to be vaccinated. It is then possible that getting the vaccine relieved those fears, which may be the channel through which vaccination improved mental health.*

**Q3:
In the introduction part, there is insufficient reasons why the authors conduct this research? The author may add some crucial information about why the readers will have benefit to know the research result.**

*We have added a paragraph in the first page of the introduction. The linkage to the literature suggested by the editor also helps to solve this issue.*

**Q4:
The authors should provide the scientific reason why the panelists complete the surveys in the 14 days interval?**

*Page 6 now includes an explanation of the rationale for the periodicity of the surveys.*

**Q5:
There is no information about the validity and reliability of the PHQ-4?**

*We now cite the validation and reliability study for the PHQ-4 measure (page 7, second paragraph)*

**Q6:
The authors stated “The ever vaccinated and never vaccinated groups differ significantly in their demographic composition as well as in baseline levels of mental health. The differences are likely a result both from the vaccine eligibility rules applicable during the period that we study as well as different levels of vaccine enthusiasm or hesitancy across demographic groups” in result section. Unfortunately, there is no explanation about the occupational background of the respondents, which may explain why they get vaccinated and never vaccinated. For instance the group of health care professionals.**

*The dataset we use does not contain occupation data, unfortunately. We agree that occupations are likely to be different across groups, and in indeed the larger proportion of health care professionals among the “ever vaccinated” may indeed at least partly explain the larger percentage of respondents with a college degree in that group. We now state that in the first paragraph of page 10.*

**Q7:
1. Table 1 the authors should clearly indicate the column label (number and percentage).
2. The authors need provide the information about the total number of the respondents in the table 1.**

*The labels in columns 1 and 2 have been added and the total number of respondents is shown in the last row.*

**Q8:
In figure 1 how the authors determined the time/date of group get vaccinated? Since the vaccination for COVID-19 started in December 23, 2020, so why the authors mentioned the interval was between March 10, 2020 and March 14, 2021?**

*We agree the statement in the note of Figure 1 was confusing. We change it so that it simply says that the Ever vaccinated are those who received a dose by March 14, and the Never vaccinated are those who had not received any dose by March 14.*

**Q9:
It was less information in discussion part that the authors may explain deeper interpretation from the result part. For instance, the comparison from each group of different educational background, and ethnics.**

*We have added a clarification to the description of the heterogeneity analysis.*

**Q10:
There was miss-typed and word redundan**t

*We revised and corrected the text for typos and redundant words.*

**Response to Journal Requirements:**

When submitting your revision, we need you to address these additional requirements.

**1. Please ensure that your manuscript meets PLOS ONE's style requirements, including those for file naming. The PLOS ONE style templates can be found at**

[https://journals.plos.org/plosone/s/file?id=wjVg/PLOSOne_formatting_sample_main_body.pdf](https://urldefense.com/v3/__https:/journals.plos.org/plosone/s/file?id=wjVg*PLOSOne_formatting_sample_main_body.pdf__;Lw!!LIr3w8kk_Xxm!4tD71uA8Z5D3u_Ulz91Y236NEQbA_lUnKuhpZVK-_zpmUZzC22XTBaufQwGLtpE$) and

[https://journals.plos.org/plosone/s/file?id=ba62/PLOSOne_formatting_sample_title_authors_affiliations.pdf](https://urldefense.com/v3/__https:/journals.plos.org/plosone/s/file?id=ba62*PLOSOne_formatting_sample_title_authors_affiliations.pdf__;Lw!!LIr3w8kk_Xxm!4tD71uA8Z5D3u_Ulz91Y236NEQbA_lUnKuhpZVK-_zpmUZzC22XTBauf6WMN9pQ$)

**2. Please include additional information regarding the survey or questionnaire used in the study and ensure that you have provided sufficient details that others could replicate the analyses. For instance, if you developed a questionnaire as part of this study and it is not under a copyright more restrictive than CC-BY, please include a copy as Supporting Information.**

*The surveys are publicly available at* [*https://uasdata.usc.edu/page/Covid-19+Documentation*](https://uasdata.usc.edu/page/Covid-19+Documentation)*. We have added the link to the study.*

3**. We note you have included a table to which you do not refer in the text of your manuscript. Please ensure that you refer to Table 1 and 3 in your text; if accepted, production will need this reference to link the reader to the Table.**

*We have added the reference to Tables 1 and 3 in the text.*

**4. Please review your reference list to ensure that it is complete and correct. If you have cited papers that have been retracted, please include the rationale for doing so in the manuscript text, or remove these references and replace them with relevant current references. Any changes to the reference list should be mentioned in the rebuttal letter that accompanies your revised manuscript. If you need to cite a retracted article, indicate the article’s retracted status in the References list and also include a citation and full reference for the retraction notice.

[Note: HTML markup is below. Please do not edit.]**
